# Undue reliance on *I*^2 ^in assessing heterogeneity may mislead

**DOI:** 10.1186/1471-2288-8-79

**Published:** 2008-11-27

**Authors:** Gerta Rücker, Guido Schwarzer, James R Carpenter, Martin Schumacher

**Affiliations:** 1 Institute of Medical Biometry and Medical Informatics, University Medical Center Freiburg, Germany; 2 German Cochrane Centre, University Medical Center Freiburg, Germany; 3 Medical Statistics Unit, London School of Hygiene and Tropical Medicine, London, UK

## Abstract

**Background:**

The heterogeneity statistic *I*^2^, interpreted as the percentage of variability due to heterogeneity between studies rather than sampling error, depends on precision, that is, the size of the studies included.

**Methods:**

Based on a real meta-analysis, we simulate artificially 'inflating' the sample size under the random effects model. For a given inflation factor *M *= 1, 2, 3,... and for each trial *i*, we create a *M*-inflated trial by drawing a treatment effect estimate from the random effects model, using $s_{i}^{2}$/*M *as within-trial sampling variance.

**Results:**

As precision increases, while estimates of the heterogeneity variance *τ*^2 ^remain unchanged on average, estimates of *I*^2 ^increase rapidly to nearly 100%. A similar phenomenon is apparent in a sample of 157 meta-analyses.

**Conclusion:**

When deciding whether or not to pool treatment estimates in a meta-analysis, the yard-stick should be the clinical relevance of any heterogeneity present. *τ*^2^, rather than *I*^2^, is the appropriate measure for this purpose.

## Background

In meta-analysis, three principal sources of heterogeneity can be distinguished. These are (i) *clinical baseline heterogeneity *between patients from different studies, measured, e.g., in patient baseline characteristics and not necessarily reflected on the outcome measurement scale; (ii) *statistical heterogeneity*, quantified on the outcome measurement scale, that may or may not be clinically relevant and may or may not be statistically significant, and (iii) *heterogeneity from other sources*, e.g. design-related heterogeneity. In this article, we only deal with statistical heterogeneity. References [1-7] give an introduction to the large literature in this area. We do not discuss how to assess clinical baseline heterogeneity.

In this paper, we show that *I*^2 ^increases with the number of patients included in the studies in a meta-analysis. In the light of this, we argue that *I*^2 ^is in general of limited use in assessing clinically relevant heterogeneity.

The article is structured as follows. After introducing existing measures of heterogeneity in meta-analysis and discussing their properties, we illustrate the problem of interpreting the measure *I*^2 ^using an example from the literature. We then present a simulation study which explores the effect of sample size inflation on *I*^2^, and finally conclude with a discussion.

## Methods

Let *k *be the number of studies in a meta-analysis. Further, let *x*_*i *_be the within-study treatment effect estimate (e.g., a log odds ratio), $s_{i}^{2}$ the within-study variance of *x*_*i*_, and *w*_*i *_the weight of study *i *(*i *= 1,..., *k*). In this article, we always use inverse variance weights, that is, *w*_*i *_= 1/$s_{i}^{2}$ if the fixed effect model is used, and *w*_*i *_= 1/($s_{i}^{2}$ + *τ*^2^) if the random effects model is used (see below for definition and estimation of the heterogeneity variance *τ*^2^). Several measures of statistical heterogeneity are widely used:

1. Cochran's *Q *statistic, which under the null hypothesis of no heterogeneity follows a *χ*^2 ^distribution with *k *- 1 degrees of freedom [8]. Q is given by

$$Q=\sum_{i=1}^{k}w_{i}{\left(x_{i}-\frac{\sum w_{j}x_{j}}{\sum w_{j}}\right)}^{2};$$

2. Higgins' and Thompson's *I*^2^, derived from Cochran's *Q *by defining [4]

$$I^{2}=\max\left\{0,\frac{Q-(k-1)}{Q}\right\};$$

3. the between-study variance, *τ*^2^, as estimated in a random effects meta-analysis. There are several proposals for estimating *τ*^2 ^in a meta-analysis, such as the REML estimator or the Hedges-Olkin estimator [5-7,9]. Nevertheless, most reviewers use the moment-based estimate of *τ*^2 ^[10], implemented in RevMan [11] and calculated as

$${\hat{\tau }}^{2}=\max\left\{0,\frac{Q-(k-1)}{\sum w_{i}-\frac{\sum w_{i}^{2}}{\sum w_{i}}}\right\};$$

4. *H*^2^, derived from Cochran's *Q *by defining [4]

$$H^{2}=\frac{Q}{k-1},$$

and

5. *R*^2^, similar to *H*^2 ^and calculated from *τ*^2 ^and a so-called 'typical' within-study variance *σ*^2 ^(which must be estimated), and defined as:

$$R^{2}=\frac{\tau^{2}+\sigma^{2}}{\sigma^{2}}.$$

As seen here, and described elsewhere [4], some measures are directly related, and others approximately related. Table 1 shows key properties of the various measures; more details are given in [4]. In summary:

**Table 1:** Properties of measures of heterogeneity.

Measure	measured on		increasing with	
	scale	range	number of studies in meta-analysis	precision (size of studies)

*Q*	absolute	[0, ∞)	yes	yes
*I*^2^	percent	[0, 100%]	no	yes
*τ*, *τ*^2^	outcome	[0, ∞)	no	no
*H, H*^2^	absolute	[1, ∞)	no	yes
*R, R*^2^	absolute	[1, ∞)	no	yes

1. *Q*, which follows a *χ*^2 ^distribution with *k *- 1 degrees of freedom under *H*_0_, is the weighted sum of squared differences between the study means and the fixed effect estimate. It always increases with the number of studies, *k*, in the meta-analysis.

2. In contrast to *Q*, the statistic *I*^2 ^was introduced by Higgins and Thompson [4] as a measure independent of *k*, the number of studies in the meta-analysis. *I*^2 ^is interpreted as the percentage of variability in the treatment estimates which is attributable to heterogeneity between studies rather than to sampling error.

3. *τ*^2 ^describes the underlying between-study variability. Its square root, *τ*, is measured in the same units as the outcome. Its estimates do not systematically increase with either the number, or size, of studies in a meta-analysis.

4. *H*^2 ^is a test statistic. It describes the relative difference between the observed *Q *and its expected value in the absence of heterogeneity. Thus it does not systematically increase with the number of studies [4]. *H *corresponds to the residual standard deviation in a radial (Galbraith) plot [12]. *H *= 1 indicates perfect homogeneity.

5. *R*^2 ^is the square of a statistic *R *which describes the inflation of the random effects confidence interval compared to that from the fixed effect model. It does not increase with *k*. *R*^2 ^= 1 indicates perfect homogeneity [4].

Notice that, in contrast to *τ*^2^, the measures *Q*, *I*^2^, *H *and *R *all depend on the precision, which is proportional to study size [13]. Thus, given an underlying model, if the study sizes are enlarged, the confidence intervals become smaller and the heterogeneity, measured (say) using *I*^2^, increases. This is reflected in the interpretation: As *I*^2 ^is the percentage of variability that is due to between-study heterogeneity, 1 - *I*^2 ^is the percentage of variability that is due to sampling error. When the studies become very large, the sampling error tends to 0 and *I*^2 ^tends to 1. Such heterogeneity may not be clinically relevant.

We now explore this further using simulation. Note first that simply looking at the effect of scaling up all sample sizes by a common factor (leaving their treatment effects unchanged) is not appropriate. This is because if study sizes were truly to increase, estimates would approach the true value for each study and not be fixed at the original observed value. Instead, we simulate under the random effects model. Under this model, *μ *and *τ*^2 ^are assumed constant, and the total variance in study *i *is $\sigma_{i}^{2}$ + *τ*^2^, which decreases with increasing study sample size, eventually tending to *τ*^2^.

### Study size inflation based on the random effects model

Suppose in a meta-analysis trial *i *reports treatment effect estimate *x*_*i *_(e.g., on the log odds scale) with observed sampling variance $s_{i}^{2}$. Let *τ*^2 ^denote the heterogeneity variance. The model is

$$\begin{array}{cc} x_{i}=\mu +\sqrt{\sigma_{i}^{2}+\tau^{2}}\epsilon_{i}, & \epsilon_{i}\sim N(0,1), \end{array}$$

where *μ *is the average treatment effect. For a given inflation factor *M *= 1, 2, 3,..., the model with inflated sample size (corresponding to an *M*-fold increase in precision) is

$$\begin{array}{cc} x_{M,i}=\mu +\sqrt{\sigma_{i}^{2}/M+\tau^{2}}{\epsilon^{\prime }}_{i}, & {\epsilon^{\prime }}_{i}\sim N(0,1). \end{array}$$

We generate an illustrative meta-analysis for each inflation factor. For each trial in each meta-analysis, we generate a random *M*-inflated trial by drawing a treatment effect estimate *x*_*M*,*i *_from this model, using $s_{i}^{2}$/*M *as the within-trial sampling variance and the DerSimonian-Laird estimate ${\hat{\tau }}^{2}$ for the heterogeneity parameter *τ*^2^.

## Results

We use data from a large meta-analysis (of 70 trials) to estimate the effect of thrombolytic therapy in acute myocardial infarction [14]. The original analysis using the fixed effects model (Mantel-Haenszel method) gives an odds ratio of 0.747 with a 95% confidence interval (95% CI) of [0.705; 0.792]. Using the random effects model, the odds ratio is 0.732, 95% CI [0.664; 0.808]. The DerSimonian-Laird estimate of *τ*^2 ^is 0.018 (*H *= 1.11, 95% CI [1; 1.29], *I*^2 ^= 18.6%, 95% CI [0%; 40.1%]). As *Q *= 85, *p *= 0.0953, there is no evidence of heterogeneity.

**Figure 1:**
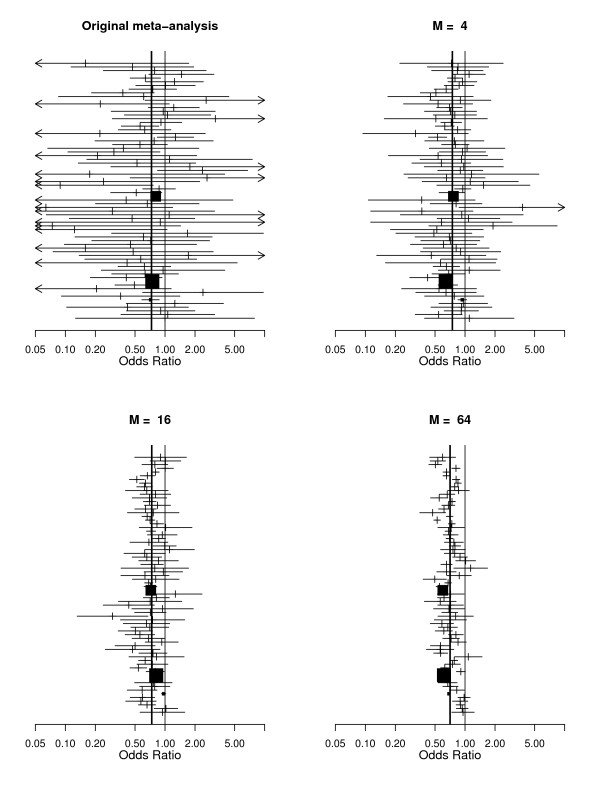
**Top left panel: Meta-analysis of thrombolytic therapy in acute myocardial infarction **[14]. Other plots: illustrative randomly sampled versions of the same meta-analysis with sample-size inflation factors of *M *= 4, 16 and 64 (details in text).

We now explore the effect of increasing *M*. Figure 1 shows forest plots of the original meta-analysis along with illustrative meta-analyses generated for *M *= 4, 16 and 64. The behavior of the heterogeneity measures is shown in Table 2. It is clear that while the variation in *τ*^2 ^is essentially random, the values of *Q*, *H *and *I*^2 ^increase rapidly with increasing sample size.

**Table 2:** Effect of increasing within trial precision (factor *M*) on heterogeneity measures (data in [14]).

Factor	Measure			
*M*	${\hat{\tau }}^{2}$	*Q *(*P*-value)	*I*^2^	*H*

1	0.018	85 (0.0953)	18.6% [0%; 40.1%]	1.11 [1; 1.29]
4	0.008	98 (0.0135)	29.2% [4.5%; 47.6%]	1.19 [1.02; 1.38]
16	0.027	454 (<0.0001)	84.8% [81.4%; 87.5%]	2.56 [2.32; 2.83]
64	0.028	1708 (<0.0001)	96.0% [95.4%; 96.5%]	4.98 [4.65; 5.32]

**Figure 2:**
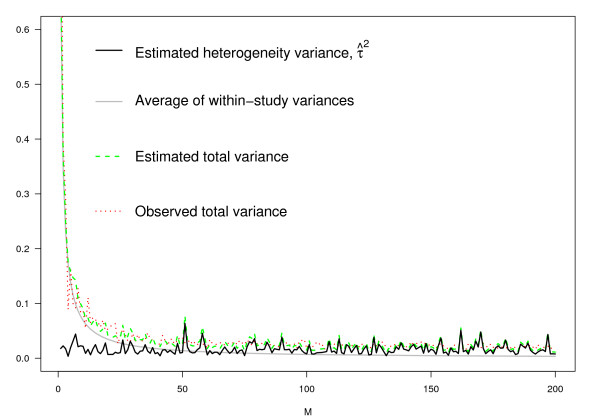
**Within-study variation, decreasing with increasing sample size while heterogeneity remains constant**. Details in text.

**Figure 3:**
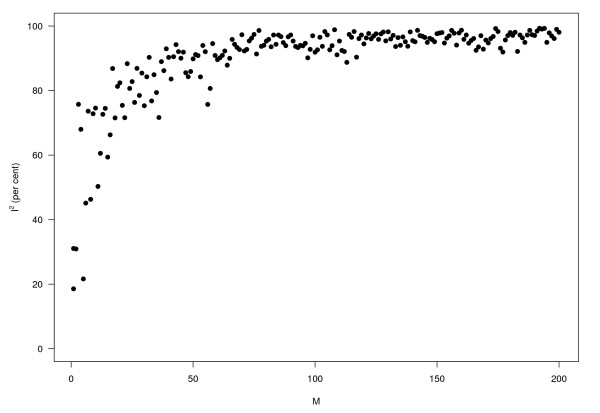
**Percentage***** I***^**2 **^**of variation due to heterogeneity rather than to sampling error against sample size (same simulation data as in Figure 2)**.

Figures 2 and 3 give two other perspectives on this. Figure 2 shows that as *M *increases, *τ*^2 ^varies randomly, while (i) the average of the within study variances; (ii) the estimated total variance (under the model), and (iii) the observed total variance, all decrease rapidly with increasing *M*. Using the same data, Figure 3 shows how *I*^2 ^behaves. Note how rapidly it approaches 100%.

### Empirical evaluation: a sample of meta-analyses

In order to examine the behavior and the order of magnitude of *I*^2 ^empirically, we further looked at a sample of 157 meta-analyses with binary endpoints. This data set was kindly provided by Peter Jüni [15]. We calculated *τ*^2 ^and *I*^2 ^for each meta-analysis. Further, for each meta-analysis, we calculated the median study size of the contributing studies, denoted *n*_*i*_, *i *= 1,..., 157. After excluding all meta-analyses with both *τ*^2 ^= *I*^2 ^= 0 (*n *= 58), we fitted a linear model to the remaining 99 meta-analyses with *I*^2 ^as outcome and ${\hat{\tau }}_{i}$ and log *n*_*i *_as covariates (thus implicitly assuming a log-normal distribution for study size).

**Figure 4:**
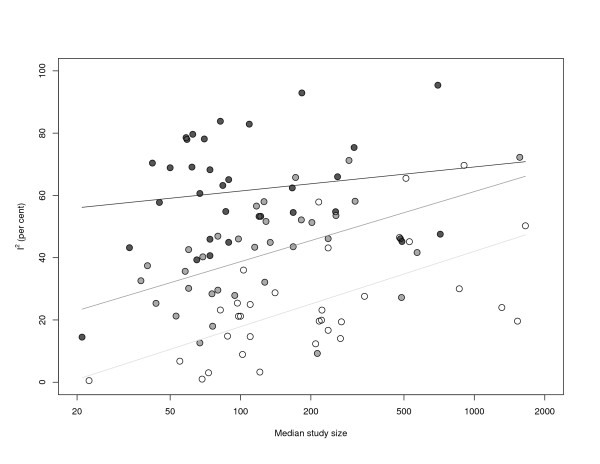
***I***^**2 **^**against median study size in a sample of 157 meta-analyses**. Light, grey and black dots and regression lines correspond to the first, second and third tercile of the distribution of *τ*^2^.

As expected, *I*^2 ^increases with both heterogeneity (*β*_*τ *_= 65.873, SE = 4.788, *p *= 0.000) and median study size (*β*_log *n *_= 8.503, SE = 1.460, *p *= 0.000). The residual standard error is 13.07 with an adjusted $R_{adj}^{2}$ = 0.6621 (*F *= 97.01, *df *= 96, *p *= 0.000). That is, even after adjusting for between-study variance *τ*^2^, *I*^2 ^depends strongly on study size. Figure 4 illustrates the results.

Light, grey and black dots and regression lines correspond to the first, second and third tercile of the distribution of *τ*^2^. Within each class of meta-analyses, *I*^2 ^is increasing with median study size.

## Discussion

The main advantage of the statistic *I*^2 ^is that it does not depend on the number of studies in a meta-analysis. Thus, using *I*^2 ^instead of *Q*, it is possible to compare the statistical heterogeneity of meta-analyses with different numbers of studies [4]. Also, *I*^2 ^is easily interpreted by clinicians as the percentage of variability in the treatment estimates which is attributable to heterogeneity between studies rather than to sampling error.

However, an immediate (but often overlooked) consequence of this interpretation is that *I*^2 ^increases with the number of patients included in the studies in a meta-analysis. In a recent simulation using continuous outcomes, others found empirically that *I*^2 ^increased with increasing numbers of patients per trial though *τ*^2 ^was kept fixed [16]. Unfortunately, as demonstrated by a recent empirical study [17], reviewers seem to be unaware of this when they use *I*^2 ^to decide whether to pool studies in a meta-analysis. Some authors also seem to be reluctant to call *I*^2 ^a statistic, using instead words such as metric [18], index [19], or even point estimate [17,18,20]. On the other hand, the term 'statistical test' is used in connection with *I*^2 ^in one of these references [20], p. 915. In another reference [18], the authors proposed an algorithm for a sensitivity analysis that successively excludes 'outlying' trials until *I*^2 ^falls below a prespecified level. In response to this [21], Higgins showed that the exclusion of a large trial with its effect close to the pooled estimate can be the most efficient way to reduce *I*^2^.

Our simulation highlights the problem of interpreting heterogeneity measured by *I*^2 ^as clinical heterogeneity. This is analogous to interpreting statistically significant effects (*P *< 0.05) as clinically relevant. In our view the decision on whether or not to pool studies in a meta-analysis should not solely be based on *I*^2^. Instead, studies with relatively large *I*^2 ^may usefully be pooled when the clinically relevant heterogeneity (in efficacy and covariates) is acceptably small.

Further, as *τ *is measured on the same scale as the outcome, it can be directly used to quantify variability. Indeed, clinically meaningful heterogeneity on the outcome scale could be pre-specified. Thus, in advance a reviewer may decide that three studies with odds ratios of 0.8, 1 and 1.25 cannot be pooled; in other words the relative effect ratios of 0.8 = 1/1.25 are too great. This corresponds to a standard deviation *τ*_0 _= - log 0.8 = log 1.25 = 0.22 = $\sqrt{0.05}$ on the log scale and thus a threshold of $\tau_{0}^{2}$ = 0.05 for the heterogeneity variance *τ*^2^.

While Higgins and Thompson in their papers [4,22] thoroughly described the properties of the various measures and distinguished between them, we feel current guidelines are likely to let misconceptions persist. For example, the 'Cochrane Handbook for Systematic Reviews of Interventions' (outdated Version 4.2.6, page 138) stated 'A value [of *I*^2^] greater than 50% may be considered as substantial heterogeneity'. The recent Version 5.0.1, while admitting that 'thresholds for the interpretation of *I*^2 ^can be misleading, since the importance of inconsistency depends on several factors', nevertheless lists overlapping ranges of *I*^2 ^which provide 'a rough guide to interpretation' (see Table 3) [23]. The result is that some reviewers conclude that studies must not be pooled if *I*^2 ^> 50% [24,25]. By contrast, Section 9.5.4 of the handbook states 'The choice between a fixed-effect and a random-effects meta-analysis should never be made on the basis of a statistical test of heterogeneity'. Further some methodologists discourage reviewers from using tests for funnel plot asymmetry if *I*^2 ^> 50% [26].

**Table 3:** Ranges for interpretation of *I*^2 ^following the Cochrane Handbook for Systematic Reviews of Interventions (Version 5.0.1) [23].

0% to 40%	might not be important
30% to 60%	may represent moderate heterogeneity
50% to 90%	may represent substantial heterogeneity
75% to 100%	considerable heterogeneity

We believe the interpretation issues stem from the concept of *I*^2 ^as 'the proportion of variance (un)explained', referred to as 'widely familiar' to clinicians by Higgins and Thompson [4] (Section 4). However, there is a fundamental difference between the interpretation of the coefficient of determination $R_{reg}^{2}$ in regression analysis, which is sub-consciously invoked by this phrase, and that of *I*_2_: On the one hand, $R_{reg}^{2}$ (that is, the square of the correlation coefficient) is a measure of the association between the dependent and the independent variable, which homes in on the true value as the sample size increases. However, *I*^2 ^tends to 100% as the number of patients increases. Although one may argue that the 'unit' corresponding to the 'observation' in a regression is the study, not the patient, this link is only strictly valid if sample size of new studies are distributed similarly to those of existing studies. This is not universally true. Often small trials are followed by larger ones. Thus *I*^2 ^will tend to increase artificially as evidence accumulates.

To address this, more weight should be given to often overlooked comments by Higgins and Thompson, [4], p 1545, who state 'Note that we do not propose that our measure should be independent of the precisions of estimates observed in the studies. Thus sets of studies with identical heterogeneity *τ*^2^, but with different degrees of sampling error *σ*^2^, will produce different measures.... Describing the underlying between-study variability ... can best be achieved simply by estimating the between-study variance, *τ*^2^.'

## Conclusion

When deciding whether or not to pool treatment estimates in a meta-analysis, the yard-stick should be the clinical relevance of any heterogeneity present. *τ*^2^, rather than *I*^2 ^is the appropriate measure for this purpose.

## Competing interests

The authors declare that they have no competing interests.

## Authors' contributions

GR proposed the model for sample size inflation, did all calculations and wrote the first draft of the manuscript. GS, JC and MS contributed to the writing and approved the final version.

## Pre-publication history

The pre-publication history for this paper can be accessed here:


